# Treating patients with geographic atrophy: are we there yet?

**DOI:** 10.1186/s40942-023-00493-6

**Published:** 2023-11-20

**Authors:** Bani Antonio-Aguirre, J. Fernando Arevalo

**Affiliations:** Wilmer Eye Institute, Johns Hopkins School of Medicine, 600 N Wolfe St; Maumenee 713, Baltimore, MD 21287 USA

**Keywords:** Age-related macular degeneration, Pegcetacoplan, Avacincaptad pegol, Complement cascade inhibitors, Multimodal imaging

## Abstract

Geographic atrophy (GA) is a progressive degenerative disease that significantly contributes to visual impairment in individuals aged 50 years and older. The development of GA is influenced by various modifiable and non-modifiable risk factors, including age, smoking, and specific genetic variants, particularly those related to the complement system regulators. Given the multifactorial and complex nature of GA, several treatment approaches have been explored, such as complement inhibition, gene therapy, and cell therapy. The recent approval by the Food and Drug Administration of pegcetacoplan, a complement C3 inhibitor, marks a significant breakthrough as the first approved treatment for GA. Furthermore, numerous interventions are currently in phase II or III trials, alongside this groundbreaking development. In light of these advancements, this review provides a comprehensive overview of GA, encompassing risk factors, prevalence, genetic associations, and imaging characteristics. Additionally, it delves into the current landscape of GA treatment, emphasizing the latest progress and future considerations. The goal of starting this discussion is to ultimately identify the most suitable candidates for each therapy, highlight the importance of tailoring treatments to individual cases, and continue monitoring the long-term implications of these emerging interventions.

## Introduction

Age-related macular degeneration (AMD) is recognized as a prominent contributor to significant vision impairment in individuals aged 50 years and above [1]. The condition can be categorized based on the size of the drusen and retinal pigment epithelium (RPE) abnormalities, ranging from early to intermate and late AMD [2]. The rate of progression from early or intermediate AMD to advanced disease can vary significantly, ranging from 0.4 to 53% over a 5 year period [3]. This variation is influenced by factors such as the severity and monocular or binocular involvement of the disease [4, 5]. Moreover, AMD can also be classified into two distinct phenotypes: exudative and non-exudative forms [6]. The late or advanced stage of these phenotypes are commonly referred to as neovascular AMD (nvAMD) and geographic atrophy (GA), respectively [2, 7].

Geographic atrophy (GA) is characterized by either complete or partial depigmentation of the RPE, along with well-defined atrophic lesions in the outer retina [7]. These lesions occur due to the loss of photoreceptors, RPE, and the underlying choriocapillaris. Initially, these lesions tend to appear in the perifoveal region while sparing the central area, but they progressively enlarge and merge over time, ultimately affecting the fovea [5, 8]. Subjects with foveal involvement experience severe central visual acuity (VA) decline, while those with parafoveal or foveal sparing lesions may have preserved VA but difficulties with contrast sensitivity and reading [9]. The median time from non-central GA to central GA is estimated to be approximately 2.5 years [10]. Furthermore, slightly over 40% of GA patients are classified as legally blind (VA 20/200 or worse) [11, 12]. Advanced AMD has been associated with a significant (63%) reduction in the average patient's quality of life (QoL), a magnitude comparable to the impact of a catastrophic stroke or end-stage cancer on QoL [13].

The pathophysiology of GA is complex and multifactorial. According to the current hypotheses, GA is initiated by the accumulation of oxidative stress, which can be attributed to aging, environmental factors, and other causes [14, 15]. This oxidative stress triggers inflammation through multiple pathways, including the complement cascade [16]. However, when regulatory components within these pathways are compromised, as seen in certain genetic risk factors associated with GA, chronic inflammation can eventually lead to the characteristic death of the retinal cells observed. Consequently, complement inhibition has emerged as a prominent candidate for therapeutic intervention in the treatment of GA.

The upcoming years hold great potential for transformative advancements in the treatment of GA. A significant milestone was reached on February 17, 2023, when the Food and Drug Administration (FDA) granted approval to pegcetacoplan (Syfovre, Apellis Waltham, Massachusetts, U.S.), marking the first-ever approved treatment for GA [17, 18]. Alongside this breakthrough, two other drugs in phase III trials, avacincaptad pegol (Zimura, Iveric Bio, New Jersey, U.S.) and ALK-001 (Alkeus Pharmaceuticals, Cambridge, Massachusetts, U.S), were granted breakthrough therapy status from the FDA [19–21]. In August 5, 2023 Astellas Pharma Inc announced avacincaptad pegol intravitreal solution, now commercialized as IZERVAY^™^, has received FDA approval for the treatment of GA [22]. These recent developments in research and clinical trials have kindled optimism for the development of interventions capable of slowing down or halting the progression of GA. With the emergence of novel therapeutic approaches and the FDA’s approval of innovative drugs, the question arises: Are we finally at a point where effective treatment strategies for GA are within our reach? This review aims to provide an overview of the present understanding of GA, encompassing risk factors, genetic associations, and imaging characteristics. Furthermore, we will delve into the current landscape of GA treatment, emphasizing the latest breakthroughs and future considerations.

## Risk factors

AMD is a complex and multifactorial disease with several risk factors associated with its development. Age and smoking are two factors strongly associated with the diagnosis of any AMD phenotype. Age, in particular, is the most significant factor associated with any AMD diagnosis [23]. Numerous studies have consistently demonstrated that the prevalence of AMD rises with advancing age. Overall, the risk of developing AMD escalates by 4.2 times with each passing decade [24]. A comprehensive analysis pooling data from three large cohort studies revealed a substantial rise in AMD risk in older individuals. Specifically, the risk of AMD increased more than eight-fold in those aged 70–79 years and soared over 30-fold in those aged 80 to 86 years, compared to those aged 55–69 [23]. Although these cohorts included mostly White patients of Northern European ethnic origin this association remains constant across multiple geographic regions and ethnicities [25]. Another non-modifiable risk factor for AMD is a positive family history in a first-degree relative [26]. In addition, the high concordance of AMD observed in monozygotic twins, as compared to dizygotic twin pairs, strongly suggests a genetic component to the disease [27].

Smoking, on the other hand, stands out as the most strongly associated modifiable risk factor for AMD. Individuals who smoke more than 25 cigarettes per day are twice as likely to develop AMD compared to non-smokers [28]. Even ex-smokers are at increased risk, and this does not seem to decrease significantly after quitting [23, 28]. Obesity is another potentially modifiable risk factor associated with late AMD and progression from early to advanced stages [29, 30]. While factors such as hypertension and cardiovascular disease may contribute to the risk of AMD, the association is inconsistent across observational studies [25, 31]. Some observational studies have found that factors such as female sex and history of cataract surgery are more strongly associated with nvAMD [24, 25]. However, it is important to mention that a systematic review of randomized clinical trials did not find an association between cataract surgery and development of nvAMD or GA progression 12 months after cataract surgery [32]. Additional prospective studies are required to assess the long-term impact of cataract surgery on AMD.

## Prevalence

AMD poses a significant global burden, accounting for a substantial portion of blindness cases worldwide, with a prevalence rate of 8.7% [33]. Estimates indicate that over 5 million individuals currently have advanced AMD, and projections suggest that this number will exceed 10 million by 2040 [1]. Irrespective of geographic region and ethnicity, an age-dependent prevalence association remains constant.

However, in terms of ethnic variation in AMD, individuals with European ancestry exhibit the highest prevalence rates [24, 33]. In the United States, it is estimated that GA affects approximately 0.28% of individuals aged 60–64, with an increase to 0.98% among those aged 70–74 [34]. Conversely, the prevalence of GA among the Asian population is considerably lower. Among this population, the estimated prevalence of GA is 0.09% among individuals aged 60–69, and it increases to 0.29% among those over 70 [35]. Additionally, the rate of conversion of nvAMD to GA is significantly lower among the Asian population (1:1) compared to the rate in predominantly white individuals (1:3) [24, 35].

## Genetics

AMD is a disease with a strong genetic component. The heritability of AMD, which refers to the proportion of variability in risk attributed to genes, has been estimated to be between 45 and 70%. A comprehensive Genome-Wide Association Study of people with European and Asian ancestry identified 19 highly associated loci that could potentially explain up to 70% of the variability [36]. These genomic regions are enriched in genes involved in crucial biological processes, including the regulation of complement activity *(CFH, C2-CFB, C3, CFI*), lipid metabolism (*LIPC, CETP,* and *APOE)*, remodeling of the extracellular matrix (*HTRA1, ADAMTS9/MIR548A2*), and angiogenesis (*VEGFA)*.

Individuals with gene variants are at higher risk of developing AMD, with each variant contributing a slight to moderate increase in risk. Notably, multiple variants, mainly in *CFH* and *ARMS2,* have been associated with disease progression [37–39]. In particular, Y402H polymorphism in *CFH* has been consistently linked to an increased incidence and progression of AMD across different populations [40–42]. In contrast, other variants like Val62Ile in *CFH* and other polymorphisms in *C2* and *BF* have shown a potential protective effect [43, 44].

Furthermore, there have been reports of interactions between genetic variants and treatment, as well as the potential for multiplicative effects when these variants are present in conjunction with common risk factors like cigarette smoking [45]. Given the multifactorial and polygenic nature of AMD, several risk calculators have been developed to estimate the risk of AMD and its progression.

## Imaging: optical coherence tomography and fundus autofluorescence

Currently, multimodal imaging is recommended to assess the presence and progression of GA [46]. Among these modalities, optical coherence tomography (OCT) has proven to be a useful tool to identify biomarkers associated with its progression in patients with intermediate AMD (iAMD), as well as evaluate the macula and quantify GA lesions [47].

**Fig. 1:**
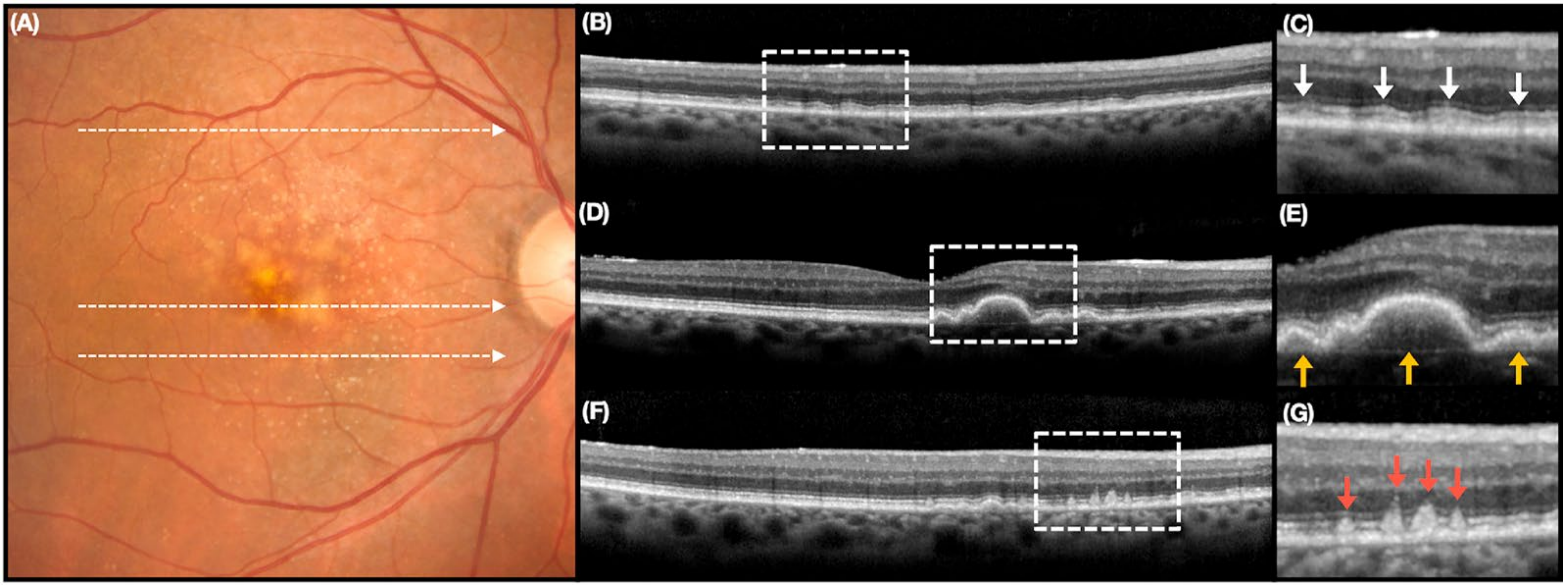
Optical Coherence Tomography (OCT) Illustration of Drusen and Reticular Pseudodrusen (RPD), **A** Color Fundus Photography depicts conventional drusen in the macular region and RPD positioned near the arcades. The OCT images correspond to the dashed lines on the fundus photography, with the third column displaying magnified views of the second column. **B** “Ribbon” RPD, hyperreflective material positioned above the retinal pigmented epithelium (RPE), leading to a modification in the contour of the ellipsoid zone (EZ). **C** Magnified view of area delineated by white dashed box, arrows indicate "Ribbon" RPD. **D** Conventional Drusen, located between the RPE and Bruch’s membrane. **E** Magnified view of area delineated by white dashed box, arrows indicate conventional Drusen. **F** “Dot” RPD, hyperreflective material penetrates through the EZ. **G** Magnified view of area delineated by white dashed box, arrows indicate "Dot" RPD. (Reprinted from Wu Z, Fletcher EL, Kumar H, Greferath U, Guymer RH. Reticular pseudodrusen: A critical phenotype in age-related macular degeneration. Progress in Retinal and Eye Research. 2022;88:101017, with permission from Elsevier)

In recent years, there has been an increased interest in identifying OCT biomarkers in iAMD that correlate with progression to advance AMD. This is mainly due to the fact that individuals with iAMD in at least one eye are estimated to be 18 times more likely to develop advanced AMD compared to those without drusen [48]. OCT plays a crucial role in this context as it enables the visualization and measurement of drusen, which are the hallmark lesions in AMD (Fig. 1D) [49]. Drusen can be classified based on their size: small (< 63 μm), intermediate (> 63 μm), and large (> 125 μm) [2]. The size and number of drusen have been associated with a higher risk of progression from iAMD to GA [50]. Notably, individuals with a drusen volume exceeding 0.03 mm^3^ are over 4 times more likely to develop GA within a 2 year period compared to those with smaller drusen [50].

Other OCT biomarkers include the presence of hyperreflective foci located over the drusen, hyporeflective foci within the drusen (calcified drusen), and the presence of reticular pseudodrusen (RPD) [51–54]. Of these, RPD are considered a distinct phenotype characterized by yellowish, interlacing deposits located in the subretinal space between the RPE and photoreceptors (Fig. 1) [55]. Evidence suggests that individuals with reticular pseudodrusen have a higher likelihood of progressing to late AMD, particularly GA [56, 57]. Furthermore, GA enlargement is significantly faster in eyes with RPD compared to those without RPD, with rates of 0.379 and 0.273 mm/year, respectively [58].

**Fig. 2:**
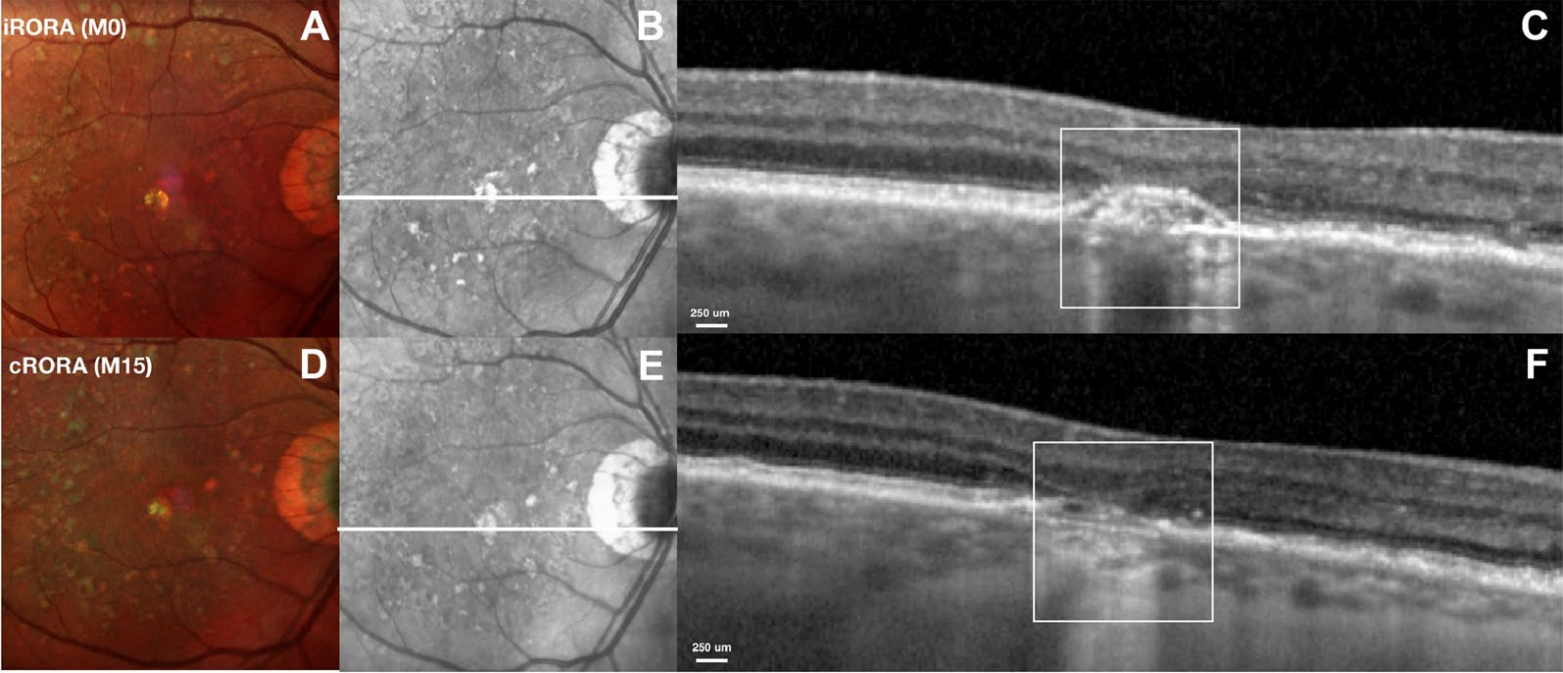
Optical Coherence Tomography Classification of Geographic Atrophy. The Classification of Atrophy Meeting (CAM) group has recommended the adoption of standardized terms such as incomplete retinal pigmented epithelium (RPE) and outer retinal atrophy (iRORA) and complete RPE and outer retinal atrophy (cRORA) in age-related macular degeneration classification. cRORA corresponds to an area of at least 250 μm on a single horizontal B-scan (Reprinted from Corradetti G, Corvi F, Nittala MG, Nassisi M, Alagorie AR, Scharf J, et al. Natural history of incomplete retinal pigment epithelial and outer retinal atrophy in age-related macular degeneration. Canadian Journal of Ophthalmology. 2021;56(5):325–34, with permission from Elsevier)

Recently, a novel classification has been developed to assess atrophic lesions in GA based on OCT parameters. The Classification of Atrophy Meeting (CAM) group has recommended the adoption of standardized terms such as incomplete RPE and outer retinal atrophy (iRORA) and complete RPE and outer retinal atrophy (cRORA) in AMD classification. cRORA corresponds to an area of at least 250 μm on a single horizontal B-scan (Fig. 2) [47, 54]. Individuals presenting with iRORA are considered at a higher risk of developing cRORA [54]. To meet the iRORA criteria, three OCT criteria must be fulfilled, including attenuation or disruption of the RPE, evidence of photoreceptor degeneration, and increased signal transmission into the choroid [54]. The CAM group also emphasizes the importance of a broader usage of the term “nascent GA,” which may be considered a subtype of iRORA and has previously been recognized as a distinct predictor of GA lesions [54, 59]. Therefore, nascent GA can be used when iRORA is present, and there is no history or evidence of macular neovascularization, indicating the initiation of progression toward GA [54].

Fundus autofluorescence (FAF) is a crucial imaging modality for evaluating GA and offers valuable structural–functional correlation [46, 60]. It enables the identification and measurement of atrophic lesions, which is important due to their association with the progression rate of GA. In GA, the atrophic lesions exhibit a loss of RPE and lipofuscin, the primary fluorophores responsible for FAF. As a result, these areas display a loss of signal or hypoautofluorescence.

**Fig. 3:**
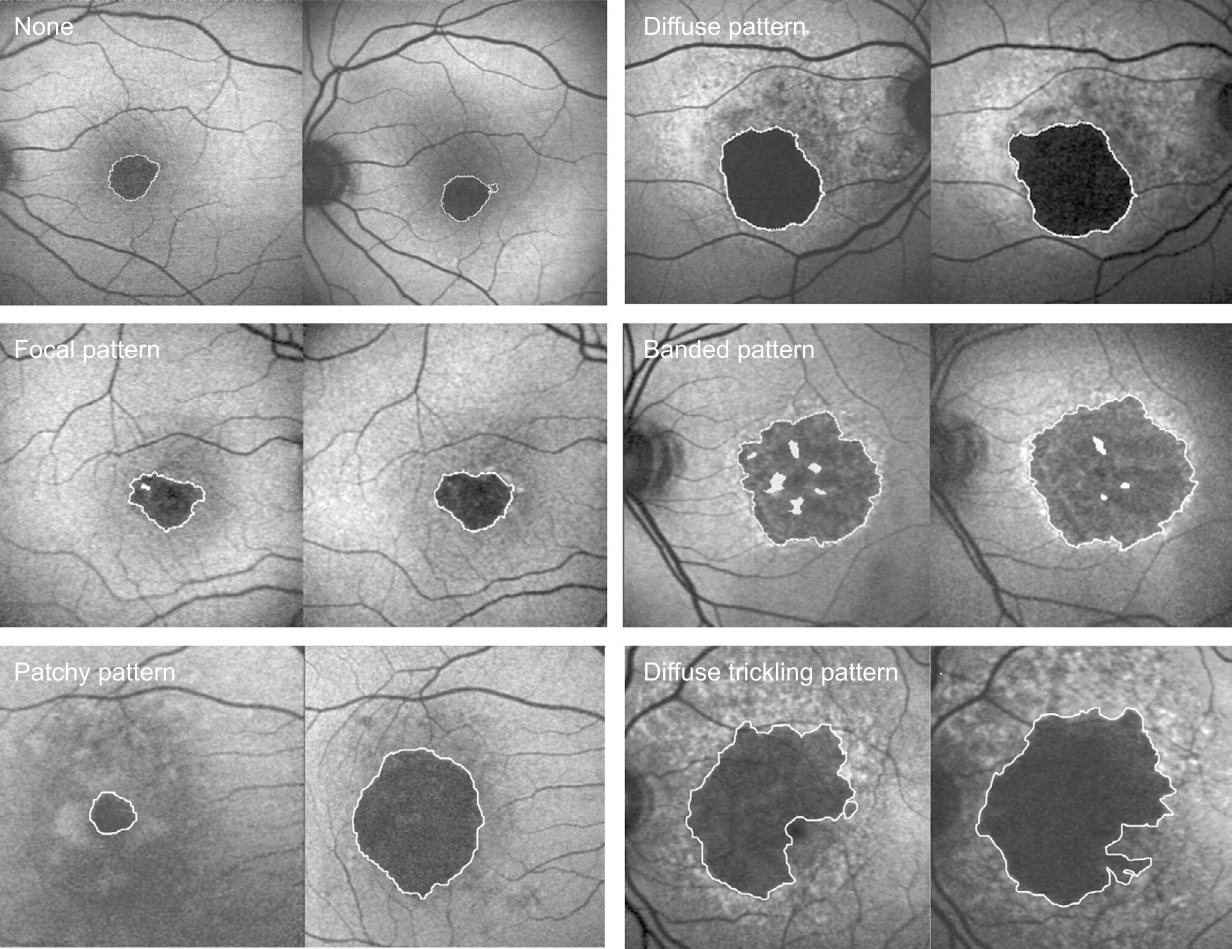
Fundus Autofluorescence Patterns of Geographic Atrophy Patterns. The Fundus Autofluorescence in Age-related Macular Degeneration (FAM) study categorized geographic atrophy eyes into five distinct phenotypes: none, focal increased, banded, patchy, and diffuse. The pattern is presented in pairs, with the baseline FAF image on the left and the follow-up FAF image on the right (follow-up ranged from 12 to 25 months). Diffuse pattern is further divided into reticular, branching, fine granular, fine granular with peripheral punctate spots and trickling. (Reprinted from Holz FG, Bindewald-Wittich A, Fleckenstein M, Dreyhaupt J, Scholl HPN, Schmitz-Valckenberg S. Progression of Geographic Atrophy and Impact of Fundus Autofluorescence Patterns in Age-related Macular Degeneration. American Journal of Ophthalmology. 2007;143(3):463–72.e2, with permission from Elsevier)

The Fundus Autofluorescence in Age-related Macular Degeneration (FAM) study categorized GA eyes into five distinct phenotypes: none, focal increased, banded, patchy, and diffuse (Fig. 3) [61]. Eyes without or with focal atrophy demonstrate the slowest progression rate, with a median progression of 0.38 and 0.81 mm^2^/year, respectively. On the other hand, eyes with banded or diffuse atrophy exhibit a higher median progression rate of 1.81 and 1.77 mm^2^/year, respectively [61].

The focality of atrophic lesions has also been linked to disease progression [62–64]. In the Beaver Dam Eye Study, eyes with multifocal lesions showed a greater increase in atrophy area (12 mm^2^ versus 2 mm^2^ over a 5 year period), a higher proportion of lesions progressing towards the foveal center (83% versus 22%), and a more significant decrease in vision (mean of 22 versus 10 letters) compared to eyes with a single GA lesion [62]. FAF is also valuable in assessing foveal involvement, as studies have revealed that extra-foveal lesions tend to progress more rapidly (2.05 mm^2^/year versus 1.28 mm^2^/year, respectively) [64]. Given the strong correlation between atrophic lesions and their prognostic factors, the GA area measured through FAF serves as the primary endpoint in most ongoing clinical trials.

## Complement cascade

**Fig. 4:**
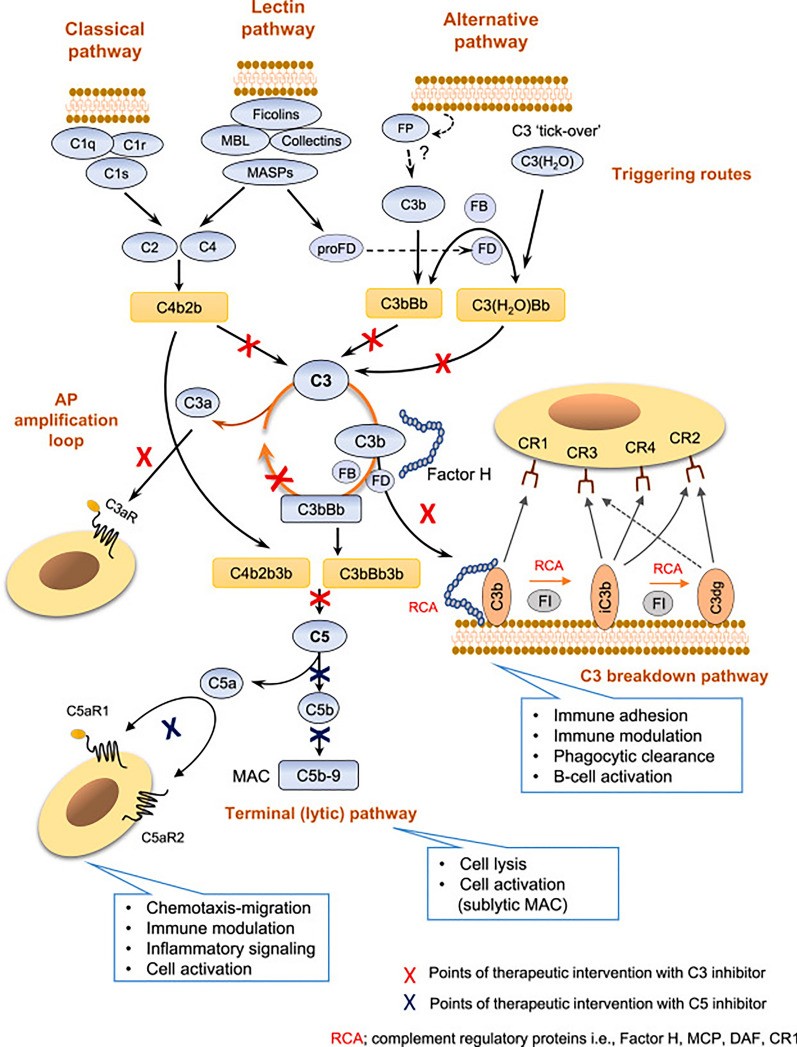
Overview and Pathways of the Complement System. The complement cascade involves a multitude of protein interactions occurring within the plasma, within the cells, and membranes. This process is initiated through the classical, lectin, and alternative pathways. These three pathways ultimately converge at C3, a pivotal mediator with diverse functions. The accompanying image highlights points of therapeutic intervention indicated by C3 (in red) and C5 (in blue) inhibition (Reprinted from Kim BJ, Mastellos DC, Li Y, Dunaief JL, Lambris JD. Targeting complement components C3 and C5 for the retina: Key concepts and lingering questions. *Prog Retin Eye Res.* 2021;83:100936, with permission from Elsevier)

The complement cascade, a complex system of proteins involved in immune response and inflammation, has emerged as a significant player in the pathogenesis of AMD. The complement system can be activated through three pathways, the classical, alternative, and lectin (Fig. 4) [65]. These pathways eventually lead to the activation of C3. Specifically, the activation of C1q, the initial component of the classical pathway, has been acknowledged as a crucial driver of complement activity in AMD. This understanding has been solidified through animal models and precise pharmacological intervention research [66]. Upon activation of these pathways, a series of proteolytic cleavage events occur, resulting in the generation of various complement components [66]. These components can have multiple effects, including the opsonization of pathogens, the recruitment and activation of immune cells (such as phagocytes), the formation of membrane attack complexes (MAC) to directly lyse target cells, and the release of pro-inflammatory mediators that promote inflammation [67, 68].

Investigations into the molecular composition of drusen provided early insights into the potential involvement of immunological factors in the disease [69]. The presence of proteins associated with inflammation and immune responses, including components of the complement system, within drusen deposits suggests an immunological component in AMD [69]. Additionally, genetic studies have identified specific variants in complement genes that regulate the system are associated with an increased risk of AMD [68].

The dysregulated complement activation in AMD leads to the formation of complement complexes and the deposition of complement components, such as C3 and C5b-9 (MAC), within retinal tissues [67]. This deposition triggers inflammation, oxidative stress, and damage to the RPE and photoreceptor cells [70]. Studies have shown that patients with iAMD and late AMD exhibit higher levels of complement activation compared to both control individuals and those with early AMD [71]. Given the significant implication of the complement cascade in the pathophysiology of AMD, many novel therapies aim to inhibit the formation of the MAC.

## Therapeutic options that attack the complement cascade in GA

### Pegcetacoplan

Pegcetacoplan (Syfovre, Apellis,Waltham, Massachusetts, U.S.) is a pegylated complement C3 inhibitor. It offers a more comprehensive blockage of both upstream and downstream inflammatory pathways when compared to C5 inhibition (Fig. 4) [72]. On the upstream front, inhibition of C3 hinders the formation of C3-derived fragments that contribute to the amplification of the alternative pathway. Downstream, it prevents the activation of the complete cascade, such as C5a, and the initiation of MAC activation. The OAKS (NCT03525613) and DERBY (NCT03525600) studies were phase III trials involving a total of 637 and 621 patients, respectively [18, 73]. These multicenter, randomized, double-masked, sham-controlled studies aimed to assess the efficacy and safety of SYFOVRE™ (pegcetacoplan injection) in patients age 60 years and older with foveal and extrafoveal GA. The primary endpoint was the change in the total area of GA lesions measured by FAF over a period of 12 months.

Both the monthly and every-other-month (EOM) administration of SYFOVRE demonstrated a significant reduction in the rate of GA lesion growth compared to sham injections. In the OAKS trial, monthly administration resulted in a 22% reduction (P < 0.0001), while EOM administration showed an 18% reduction (P = 0.0002) in GA lesion growth at 24 months [17]. In the DERBY trial, monthly administration led to a 19% reduction (P = 0.0004), and EOM administration resulted in a 16% reduction (P = 0.003) in GA lesion growth over the same duration.

In the phase II trial, a decrease in GA growth measured by FAF was observed, but no improvement in visual parameters, including best-corrected VA (BCVA), low-luminance BCVA, and low-luminance visual acuity deficit (LL-VD) was noted in individuals who received pegcetacoplan [74]. Additionally, all groups showed a similar rate of decline in VA measures and LL-VD as 12 months. New data from the phase III trial revealed that patients receiving monthly and EOM pegcetacoplan experienced less loss of retinal sensitivity measured by microperimetry and fewer new scotomas compared to the sham group [75].

In clinical trials, the administration of SYFOVRE was found to be associated with higher rates macular or choroidal neovascularization. Specifically, the incidence of nvAMD was 12% in the monthly administration group, 7% in the EOM administration group, versus 3% in the control group by month 24 [17]. These findings highlight the importance of closely monitoring patients receiving SYFOVRE for the potential development of nvAMD and timely administration of anti-VEGF therapies. Within the EOM group, retinal hemorrhages and vitreous detachments were observed at rates of 5% and 6%, respectively, compared to 4% in the monthly administration group and 3% in the sham group for both occurrences [76]. Instances of ocular inflammation, defined by the presence of conditions such as vitritis, vitreous cells, iridocyclitis, uveitis, anterior chamber cells, iritis, or anterior chamber flare, were reported in 4% of patients receiving monthly SYFOVRE and 2% of those treated with EOM. In contrast, ocular inflammation was observed in less than 1% of the sham group. There were instances of optic ischemic neuropathy reported in 1.7% of patients receiving monthly SYFOVRE, 0.2% of patients treated with EOM, and no cases reported among patients in the sham group [76].

Throughout the OAKS and DERBY trials, no instances of retinitis or occlusive/nonocclusive vasculitis were reported. However, since the launch of SYFOVRE in February 2023 until July 2023, confirmed cases of occlusive (4 cases) and non-occlusive (3 cases) retinal vasculitis have emerged [77]. These incidents have been reported to the FDA, and the company is actively engaged in further investigation, and committed to providing updates [77]. As of July 2023, approximately 68,000 vials of SYFOVRE have been distributed [77]. However, the precise number of injections administered and the total count of treated patients remain unclear (Unpublished data, ASRS Research and Safety in Therapeutics Committee presented at the American Society of Retina Specialists 41st Annual Meeting July 28-August 1, 2023. Seattle, WA).

Additionally, an extension of the Phase III trial, known as GALE (NCT04770545), has been initiated. This extension includes 80% of participants who completed the OAKS and DERBY studies. The GALE trial will continue monitoring and reporting on long-term and newly emerging adverse events [78].

### Avacincaptad pegol

Avacincaptad pegol (IZERVAY, Iveric Bio, New Jersey, U.S.). It is a modified aptamer that has been pegylated to enhance its stability and prolong its half-life. The drug functions by inhibiting the cleavage of complement factor C5 into its terminal fragments, C5a and C5b-9 (Fig. 4). This action effectively blocks the activation of the complement cascade and the subsequent formation of the MAC [79].

So far, the results from the phase II/III trial, GATHER1 study (NCT02686658), have shown the efficacy of avacincaptad pegol in reducing the progression of GA over 12 and 18 months in subjects with non-foveal GA. At 12 months, a reduction of 27.4% (P = 0.0072) was observed in the 2 mg cohort, and a similar reduction of 27.8% (P = 0.0051) was observed in the 4 mg cohort, compared to their respective sham treatment cohorts [80]. The results, at 18 months, showed a similar decrease in mean GA growth, with a reported reduction of 28.1% (P = 0.0014) reported in the 2 mg and 30.0% (P = 0.0021) in the 4 mg cohort [81].

At 18 months, patients receiving avacincaptad pegol also demonstrated better outcomes in terms of BCVA (ETDRS letters) and LLVA compared to their respective sham groups. The mean change in BCVA was − 12.7 in the 2 mg and − 4.27 in the 4 mg cohort compared to − 15.1 and − 7.07 in their respective sham groups. Meanwhile, the change in LLVA was − 2.72 for those receiving 2 mg and 2.85 in 4 mg versus − 3.10 and 1.68 corresponding sham groups [81].

It is important to note that while the treatments were generally well tolerated, a higher percentage of patients in the treatment groups developed macular neovascularization. Specifically, 11.9% in the 2 mg developed neovascularization and 15.7% in the 4 mg cohort, compared to 2.7% in the sham group [81]. Moreover, there was one instance (1.5%) of optic ischemic neuropathy in the 2 mg cohort and a case of retinal detachment (1.2%) in the 4 mg cohort. Notably, an eye that received the 2 mg dose of avacincaptad pegol developed intraocular inflammation, vitritis, during the seventh month of follow-up, which resolved by the eleventh month. Another eye that received the 4 mg dose experienced cystoid macular degeneration and was reported as a treatment-emergent adverse event [81].

Phase III, GATHER 2 Study aims to continue updating data on the efficacy and safety of avacincaptad pegol. However, the post-hoc analysis for vision loss from these pivotal studies was recently presented and signals up to a 59% reduction in the rate of vision loss with avacincaptad pegol 2 mg compared to sham treatment at 12 months. Vision loss in this analysis was defined as a loss of ≥ 15 letters (EDTRS) in BCVA from baseline measured at any two consecutive visits up to month 12. (Danzig et al., IOVS 2023; ARVO E-abstract 984) [82].

## Other therapeutic options

### Gene therapy

Gene therapy trials are currently being conducted to investigate various strategies, including inhibiting the complement cascade and enhancing regulatory proteins. One such therapy is HMR59, developed by Hemera Biosciences and Janssen Pharmaceuticals. This gene therapy is administered intravitreally, and its goal is to augment the expression of a soluble form of CD59 (sCD59) in normal retinal cells. This recombinant CD59 effectively prevents the formation of the MAC, which represents the final step in complement-mediated cell lysis. In the phase I trial (NCT03144999), 17 patients were enrolled to assess the safety and tolerability of the vector at three different dosage levels. No results from the trial have been published at this time [83].

Another therapy under investigation is GT005, developed by Gyroscope Therapeutics and Novartis. GT005 is a recombinant adeno-associated viral vector encoding CFI. In the FOCUS trial (NCT03846193), a phase I/II study, the safety and delivery methods of GT005 were evaluated (Nielsen et al., IOVS 2022; ARVO E-abstract 1504) [84]. The trial employed a dose-escalation and dose-expansion approach using transvitreal subretinal injection (TVSI) or the Orbit subretinal delivery system (Orbit SDS). The primary objective of the dose-escalation phase was to establish the maximum tolerated dose of GT005, followed by the recruitment of additional patients during the dose-expansion phase [85]. Preliminary results have shown that TVSI delivery is well-tolerated, and dose escalation did not lead to an increasing trend in reported adverse events. Most reported adverse effects so far have been mild and not directly associated with GT005. Phase II trials are currently ongoing and will continue to assess the safety profile and efficacy of the vector at 8 and 96 weeks after administration.

### Cell therapy

Most of the cell therapies for GA have primarily focused on transplanting RPE cells. In a phase I/II trial (NCT01344993) involving 9 patients, the safety and tolerability of subretinally transplanted RPE cells derived from human embryonic stem cells (hESC) were assessed [86]. After 12 months, a significant difference was observed in the number of letters read between the treated eyes and non-treated eyes (median of 14 letters versus − 1 letter; P = 0.0117) [86]. Furthermore, the treated eyes demonstrated a median improvement of 16 and 25 points in the National Eye Institute Function Questionnaire 25 at 3 and 12 months, respectively. It is worth noting that most of the reported adverse events were associated with vitrectomy or delivery procedure, as well as immunosuppression [86].

Another phase I/IIa trial (NCT02590692) assessed the feasibility of delivering a monolayer of RPE cells derived from human embryonic stem cells (hESC) using a composite implant called CPCB-RPE1 in 16 patients [87]. An interim analysis of 5 subjects who received the implant indicated integration of photoreceptors in the recipients using OCT images [88]. Notably, none of the patients who received the implant experienced further progression in vision loss (measured in ETDRS letters), and one subject exhibited a 17-letter improvement at day 60 after implantation, which was sustained at day 120 of evaluation. Importantly, no safety concerns have been reported for any of the patients who underwent the procedure and transplantation [88].

Other options to administer these products, such as cell suspension, have been explored. A phase I/II trial (NCT02286089) involving 24 patients evaluated the safety and efficacy of administering OpRegen® (RG6501), a cell-product of RPE-derived hESC. The preliminary results suggest that this treatment is well tolerated, formation of the epiretinal membrane was the most reported adverse event at 2 months following transplantation (Banin et al., IOVS 2019; ARVO E-abstract 6402). Most recently, new data showed that most patients treated with OpRegen had either improved or maintained baseline VA (+ 7.6 letters) at 12 months (Banin et al., IOVS 2023; ARVO E-abstract 2826). Currently, a phase IIa study (NCT05626114) is undergoing to evaluate retinal integrity post-treatment using OCT.

### Microcurrent stimulation

Another promising therapeutic avenue being investigated is transpalpebral microcurrent stimulation. In a pilot study encompassing 62 patients (NCT02540148), the impact of four microcurrent sessions during the initial 2 weeks after enrollment was evaluated [89]. Additionally, two extra sessions were conducted at weeks 14 and 26. The findings revealed a noteworthy average enhancement of 10.4 letters (ETDRS) at the 30 week mark within the intervention group. Importantly, this improvement exhibited statistical significance compared to the sham group (P < 0.001). No adverse events were reported during the study.

## Conclusions and future considerations

The emerging therapies for GA bring new hope to affected patients and demonstrate the rapid pace of innovation and development in this field. However, it is important to keep in mind that the long-term implications of these medications and interventions are still being monitored. While therapies aiming to block the complement cascade generally have a safe profile, we are still uncertain about the extent to which they will affect the quality of life and visual prognosis of GA patients. Currently, we know that drugs inhibiting the complement have the potential to slow or halt the progression of the disease. However, a recent meta-analysis showed inconclusive evidence regarding the ability of pegcetacoplan to slow down vision loss after evaluating the results from 242 patients [90].

Notably, patients with extrafoveal lesions who received avacincaptad pegol exhibited a greater deceleration of disease progression compared to patients in the pegcetacoplan trials, which included both subfoveal and extrafoveal lesions. This finding underscores the importance of tailoring treatments to specific lesion types for optimal outcomes. Moving forward, a crucial consideration will be determining which patients stand to benefit the most from these therapies. Another significant aspect to consider is the risk of nvAMD. It appears that the risk of neovascularization is dose dependent. Thus, it becomes imperative to establish monitoring protocols and assess patients with risk factors for the development of nvAMD, such as a history of fellow eye nvAMD and the presence of macular neovascularization at baseline.

Regarding gene therapy and cell therapy, it is crucial to address the learning curve associated with delivering subretinal therapies. Ensuring proper training and expertise in administering these treatments will be essential for their successful implementation. Additional therapies, such as microcurrent stimulation, will require further investigation to assess their long-term effects, optimal regimens, and whether they could be effectively utilized in conjunction with other therapies.

It is important to acknowledge that the effect of these novel drugs, cell therapies, and gene therapies on the progression from intermediate to late AMD remains unknown. Clinical trials have primarily focused on patients with GA. As research continues, it will be essential to explore the potential of these therapies in delaying or preventing the transition from intermediate to late-stage AMD.

In summary, while progress has been made in treating patients with GA, several crucial considerations need to be addressed. Identifying the most suitable candidates for these therapies, tailoring treatments to specific lesion types, assessing the risk of nvAMD, and understanding the impact on disease progression from intermediate to late AMD will guide future advancements in the field.

## Data Availability

Not applicable.
